# Differentiation Induction of Human Stem Cells for Corneal Epithelial Regeneration

**DOI:** 10.3390/ijms21217834

**Published:** 2020-10-22

**Authors:** Kasem Theerakittayakorn, Hong Thi Nguyen, Jidapa Musika, Hataiwan Kunkanjanawan, Sumeth Imsoonthornruksa, Sirilak Somredngan, Mariena Ketudat-Cairns, Rangsun Parnpai

**Affiliations:** 1 Embryo Technology and Stem Cell Research Center, School of Biotechnology, Institute of Agricultural Technology, Suranaree University of Technology, Nakhon Ratchasima 30000, Thailand; kasemtheera@gmail.com (K.T.); hongcn50c@gmail.com (H.T.N.); jidapa.jah@gmail.com (J.M.); tonikuya@hotmail.com (S.I.); sirilak.som@ku.th (S.S.); ketudat@sut.ac.th (M.K.-C.); 2 Medeze Research and Development Co., Ltd. 28/9 Moo 8, Phutthamonthon Sai 4 Rd., Krathum Lom, Sam Phran, Nakhon Pathom 73220, Thailand; hataiwan@medezegroup.com

**Keywords:** mesenchymal stem cell, pluripotent stem cell, differentiation, corneal epithelium

## Abstract

Deficiency of corneal epithelium causes vision impairment or blindness in severe cases. Transplantation of corneal epithelial cells is an effective treatment but the availability of the tissue source for those cells is inadequate. Stem cells can be induced to differentiate to corneal epithelial cells and used in the treatment. Multipotent stem cells (mesenchymal stem cells) and pluripotent stem cells (embryonic stem cells and induced pluripotent stem cells) are promising cells to address the problem. Various protocols have been developed to induce differentiation of the stem cells into corneal epithelial cells. The feasibility and efficacy of both human stem cells and animal stem cells have been investigated for corneal epithelium regeneration. However, some physiological aspects of animal stem cells are different from those of human stem cells, the protocols suited for animal stem cells might not be suitable for human stem cells. Therefore, in this review, only the investigations of corneal epithelial differentiation of human stem cells are taken into account. The available protocols for inducing the differentiation of human stem cells into corneal epithelial cells are gathered and compared. Also, the pathways involving in the differentiation are provided to elucidate the relevant mechanisms.

## 1. Introduction

Cornea is the transparent anterior part of the eye. Its transparency allows light to enter into the eye and the visual sensation will be initiated once the light touches the photoreceptor cells in the retina. The curvature of the cornea plays an important role in light refraction. It has a refractive power of approximately 60 diopters or two-thirds of the total eye refractive power [1]. The cornea is the first part of the eye that touches the light and it is exposed to the external environment, therefore it is easily damaged more than other parts of the eye. It can be injured by chemicals, heat, and forces from accidents. The outer surface of the cornea is covered by epithelial tissue in which the corneal epithelial cells are generated from the stem cells at the corneoscleral junction called the limbus. Limbal stem cells, the stem cells residing in the limbus, generate new corneal epithelial cells to replace the shed old cells or damaged cells to maintain the corneal epithelial population. Severe damage, severe microbial infections, or some diseases such as Stevens–Johnson syndrome and ocular cicatricial pemphigoid [2,3] may cause the depletion of limbal stem cells. Such a condition is called limbal stem cell deficiency (LSCD). Once the limbal stem cells are lost, corneal epithelial cells are unable to be generated and it will result in visual impairment and finally vision loss.

Many medical treatments have been investigated to restore the corneal epithelium. The common strategy is the transplantation of autologous epithelial cells from the contralateral cornea [4,5]. In this strategy, limbal tissue is biopsied from the healthy cornea. Then, epithelial cells are isolated and expanded in laboratory until enough cells are obtained for transplantation. Even though this procedure is effective and has high success rate of 70–80% [4], it is unable to be applied to patients with bilateral LSCD. Transplantation of allogeneic limbal epithelial cells is an optional strategy but it has a risk of graft rejection [6,7,8]. To avoid the lack of autologous limbal epithelium and high risk of graft failure from allogeneic sources, autologous stem cells become a promising option for the regeneration of corneal epithelial tissue.

With the differentiation capacity, stem cells can be induced to be corneal epithelial cells and used in medical treatments. Various kinds of stem cells have been studied for corneal epithelial tissue regeneration [9,10,11]. Mesenchymal stem cells (MSCs), which possess multipotent differentiation capacity, can be isolated from patients and used in the treatments without the concern of immune rejection [12]. By using minimally invasive procedures and uncomplicated techniques, MSCs can be obtained from diverse tissues, including bone marrow, adipose tissue, dental pulp, umbilical cord, and amniotic fluid [13,14,15]. However, it is difficult to induce MSCs from mesoderm to undergo transdifferentiation into corneal epithelial cells which originally derived from ectoderm. Pluripotent stem cells (PSCs) such as embryonic stem cells (ESCs) and induced pluripotent stem cells (iPSCs) are also of interest. They are unlimited self-renewal and have the potential to differentiate into any cell type of the adult organs [16,17]. However, complicated techniques and expensive materials are required for obtaining PSCs [18,19].

Several publications reported about the capability of MSCs and PSCs to differentiate into corneal epithelial cells [20,21,22,23,24,25]. However, the methods used in the differentiation induction are very diverse. Until now, no standard differentiation protocol is available and most published researches studied stem cells from animals. Because animal stem cells are different from human stem cells in many physiological aspects [26,27,28], the protocols developed from the studies of animal stem cells may not be suitable for human stem cells [29,30]. Therefore, herein, we review the progress of the corneal epithelial differentiation of only human stem cells. We also provide fundamental knowledge related to the differentiation methods which include the nature of corneal epithelial cells and the relevant differentiation pathways.

## 2. Corneal Epithelial Cell

The cornea consists of three cellular layers: epithelium, stroma, and endothelium [31]. Bowman’s membrane is the thin matrix layer between epithelium and stroma whereas Descemet’s membrane is the matrix layer between stroma and endothelium [32]. The corneal epithelium covers the outermost surface of the cornea and protects the cornea from pathogen invasion [33]. The corneal epithelium is derived from surface ectoderm at 5–6 weeks of gestation [34]. There are 5–6 layers of non-keratinized stratified epithelial cells [35,36] in the corneal epithelial tissue which has a thickness of approximately 50 µm [37]. The corneal epithelium consists of three types of epithelial cells: basal cells, wing cells, and superficial cells [38]. The basal cells are arranged in a single cell layer adhered to the underlying Bowman’s membrane. They have cuboidal or columnar shape and have the ability for mitotic cell division [34]. Wing cells are in the suprabasal layers covering the layer of basal cells. They are polyhedral cells stratified 1–3 layers [39]. The superficial cells cover the outer surface of cornea. They are squamous cells with flattened nuclei. Terminal differentiation of the superficial cells results in cells sloughing off into the tears [40,41].

The integrity of corneal epithelium requires the balance between cellular proliferation and differentiation. While corneal epithelial cells are continuously desquamated from the corneal surface into the tear film, the new corneal epithelial cells are continually generated from the basal cells [37,42] and the stem cells residing at the limbus, the transitional area between the cornea and the sclera [43]. The regeneration of new corneal epithelial cells is achieved by the proliferation, migration, and differentiation of the corneal stem cells. The stem cells will divide into transient amplifying cells. The transient amplifying cells, which have rapid mitotic activity, will proliferate to get enough cell number before terminal differentiation [44]. The newly generated cells from the basal cells migrate vertically to the corneal surface and those from limbal stem cells migrate centripetally to the central cornea [45]. The differentiation process requires 7–10 days before they undergo apoptosis and desquamation [34]. By the delicate control, the rate of cell proliferation equals the rate of cell desquamation. Consequently, the mass of the corneal epithelium remains constant.

The epithelial cells of cornea, limbus, and conjunctiva possess distinct characteristics. Cytokeratins (CKs), which are intermediate filaments expressed in a tissue-specific manner [46], are usually used to distinguish corneal epithelial cells from other cell types. There are about 20 types of CKs in humans [47]. The expressions of CK1–CK10 and CK13–CK20 of the corneal, limbal, and conjunctival epithelial cells were clarified by Reinstein Merjava and colleagues [37]. One cytokeratin type can be expressed in more than one cell types but CK3 and CK12 are expressed only in corneal epithelial cells and commonly used as specific markers for corneal epithelial cells [35,47,48,49,50]. Besides CKs, corneal epithelial cells at each stage of differentiation can be characterized by some specific markers. Involucrin is expressed in the differentiated superficial corneal epithelial cells. The expression of involucrin is low in the limbus but high in the central cornea [51,52]. Epidermal growth factor receptor (EGFR) is expressed at the basal layer of corneal epithelium while transglutaminase 1 (TG1) is expressed in the upper layer [53]. Connexin 43 (Cx43), a gap junction protein which is acquired during corneal epithelial differentiation, is expressed in the basal layer of the corneal epithelium but it is not expressed in the limbus [35]. Limbal epithelial cells express CK5, CK14 [54], and some putative stem cell markers such as tumor protein (p63), ATP-binding cassette transporter G2 (ABCG2), and CK19. These stem cells markers of limbal epithelial cells will gradually decrease and finally disappear when they become transient amplifying cells and migrate to the central cornea [55].

## 3. Signaling Pathways Related to Corneal Epithelial Differentiation

The connection of the activities of Wnt/β-catenin, transforming growth factor beta (TGF-β)/Nodal, and bone morphogenetic protein (BMP) signaling pathway is necessary for the formation of the embryonic murine head [56]. Moreover, the cross-linkage of retinoic acid (RA) signaling, Wnt/β-catenin, paired-like homeodomain 2 (Pitx2) is essential for early eye development [57].

**Figure 1:**
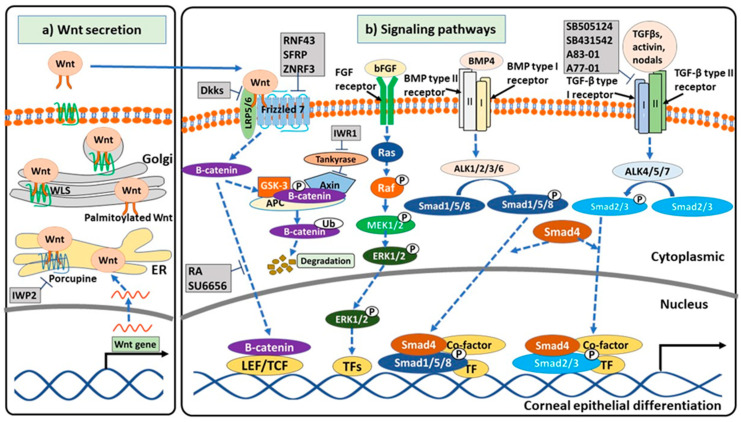
Summary of signaling pathways involved in the differentiation of pluripotent cells into corneal epithelial cells. (**a**) Inhibition of Wnt secretion: IWP2 blocks porcupine, leading to reduction of palmitoylated Wnt protein so Wnt cannot interact with Wntless (WLS) which transports Wnt to plasma membrane; (**b**) Signaling pathways in regulating the corneal epithelial differentiation of pluripotent cells. Firstly, inhibition of Wnt pathway: (1) Blocking initiation of Wnt pathway by blocking Wnt (SFRP), LRP5/6 receptor (Dkks), and Frizzled receptor (RNF43, ZNRF3, SFRP); (2) Increasing degradation of β-catenin by IWR1 lead to block transcription of Wnt target genes; (3) Decreasing translocation of β-catenin from the cytoplasm into cell nucleus by RA, SU6656 leading to decrease in expression of Wnt target genes. Secondly, the activation FGF signaling pathway by supplement bFGF. Thirdly, the activation BMP signaling pathway by using BMP4. Lastly, inhibition TGF-β signaling by using inhibitors of TGF-β type I receptor (SB505124, SB431542, A83-01, A77-01).

Wnt signaling plays an important role in eye development and its signaling is variable between the different periods [58]. Inhibiting or blocking Wnt signaling results in differentiation into epithelial cells [59,60,61]. Suppression of Wnt signaling can be done via inhibiting the initiation of Wnt signaling, stabilization, and translocation of β-catenin into cell nucleus (Figure 1). Initiation of Wnt signaling is downregulated by blocking low-density lipoprotein-related receptors 5 and 6 (LRP5/6 receptor) via Dickkopt (Dkk) proteins (Dkk1 [62,63], Dkk2 [64], Dkk3 [65]) or Frizzled 7 receptor by ring finger protein 43 (RNF43) [66], secreted Frizzled-related proteins (SFRP) [63]) or reducing Wnt secretion [67]. Reducing expression of Dkk2 via generating Dkk2-null mice increase the Wnt signaling target gene (axis inhibition protein 2 (Axin2)), leading to a decrease of corneal differentiation in ocular surface ectoderm of mouse embryo [64]. Corneal specific marker (CK12) expression is low and not continuous in the corneal epithelium of Dkk2 mutant mouse while its expression is high and continuous in wide type mouse [64]. Paired box protein (PAX6), the key transcription factor regulated eye development and maintenance of the adult cornea [41], can prevent activation of Wnt/β-catenin [58,63,68] via increasing expression of few Wnt inhibitors (Dkk1, SFRP1, SFRP2 [63], and Dkk3 [65]). Overexpression of PAX6 via its transfection into ESCs or adult stem cells induced corneal epithelium-like cells [69,70]. Two PAX6 isoforms differently induce expression of corneal specific genes (CK3 and CK12) [71]. While PAX6 isoform-a combined with Klf4 induces CK3 expression, its isoform-b combined with Kruppel-like factor 4 (Klf4) and octamer-binding transcription factor 4 (Oct4) induces CK12 expression [71]. Especially, two PAX6 isoforms combined with Klf4 and Oct4 cooperatively induce both CK3 and CK12 although induction efficiency relies on cell types [71]. Other eye field transcription factors (retinal homeobox protein (Rax), SIX homeobox 3 (Six3), homeodomain protein Optx2) also be mention that they suppress activation of Wnt signaling [58,72] but their effects on corneal differentiation have not yet reported. Another way for downregulating the initiation of Wnt signaling is inhibition of Wnt secretion by using inhibitors of porcupine, an essential enzyme for post-translational modification of Wnt, such as ETC-159, C59, IWPs, LGK974 [67]. By preventing the activity of porcupine, IWPs (IWP1, 2, 3 and 4) reduce β-catenin content in the cytoplasm [73]. Supplementation of IWP2 in low calcium medium can differentiate limbal epithelial progenitor isolated from enzymatic digestion of human limbal biopsy fragments into corneal epithelial cells expressed CK12 mRNA and protein [74]. IWP2 downregulates β-catenin content and further reduces expression of stem/progenitor markers (p63 and ABCG2). However, the effect of IWP2 on corneal epithelium differentiation depends on the culture medium and the isolated method. IWP2 increases p63 and ABCG2 expression and colony formation in the limbal epithelial progenitor cells isolated from limbal explants cultured in the supplemented hormonal epithelial medium [74].

When Wnt cannot bind with dual receptors (LRP5/6 and Frizzled 7), the level of β-catenin will be reduced by the β-catenin destruction complex which phosphorylates β-catenin, leading to ubiquitination and further degradation of β-catenin [73,75]. The β-catenin destruction complex contains glycogen synthase kinase-3 (GSK3), casein kinase 1, adenomatous polyposis coli (APC), and Axin [75]. IWR compounds (IWR 1, 2, 3, 4, 5) decrease β-catenin by upregulating β-catenin degradation via increasing the stability of Axin2 protein that is included in the β-catenin destruction complex [73]. IWR and other small molecules (XAV939, G007-LK) promote stability of Axin via inhibition Tankyrase enzyme which degrades Axin [67]. Although using of IWR1 in combination with other factors induces differentiation into corneal epithelial cells [76], using IWR1 alone does not affect lens regeneration [77].

Translocation of β-catenin is inhibited by small molecules such as RA, SU666 (the Src family kinase inhibitor), leading to a decrease in the level of β-catenin in the cell nucleus and Wnt target genes [59]. Both small molecules can drive human PSCs into simple epithelial cells that expressed CK18, CK8 [59].

The Wnt signaling pathway is blocked by deleting β-catenin or LRP5/6 receptor. Knockout β-catenin or LRP5/6 receptor can induce corneal epithelium from corneal stroma keratinocytes [60]. Knockout β-catenin or LRP5/6 receptor increase the expression of BMP4 that further enhances expression of p63, phosphorylated Smad1/5 (pSmad1/5), and phosphorylated extracellular regulated kinase 1/2 (pERK1/2), leading to corneal epithelial stratification [60].

TGF-β family members (TGF-βs, activin, and BMPs) also regulate epithelial differentiation in eye development [78]. Suppression TGF-β signaling and/or activating BMP signaling, together with another signaling (Wnt signaling or epidermal growth factor (EGF) signaling or fibroblast growth factor (FGF) signaling), are crucial for generating corneal epithelial cells from PSCs. SB505124, SB431542, A83-01, A77-01 are selective inhibitors of activin and TGF-β signaling pathway. These small molecules inhibit cytoplasmic signal transducers (Smad2 and Smad3) via inhibition of TGF-β type I receptors (activin receptor-like kinases (ALKs): ALK4, ALK5, and ALK7) [79,80,81] but SB505124 and SB431542 do not inhibit other receptors (ALK1, ALK2, ALK3, and ALK6) involved BMP signaling [79,80] while A83-01 has a slight effect on BMP receptor [81]. SB431542 supplementation improved the proliferation and maintenance phenotype of mouse lens epithelial cells [82]. Moreover, SB431542 treatment promotes the BMP activity of non-responsive human induced pluripotent stem cell line [83]. BMP signaling plays an important role in lens epithelial differentiation [78]. BMP4 or BMP7 treatment promotes the phosphorylation of Smad1/5/8 and further enhance BMP signaling [84]. Blocking TGF-β signaling and enhancing BMP and FGF signaling induce corneal limbal stem cells [85,86,87]. Treatment with blebbistatin (a non-myosin II inhibitor) at day 1, SB505124 (an inhibitor of TGF-β signaling) and basic FGF (bFGF, an activator of FGF signaling) at day 2, and BMP4 (an activator of BMP signaling) at day 3–4 drive human PSCs into surface ectodermal cells that will further differentiate into corneal limbal stem cells expressed p63, CK15 [85], ABCG2, Leucin-rich repeat-containing G-protein couple receptor 5 (LGR5), CD200 [87]. Moreover, these cells express CK3 (a specific corneal marker) after stratification culture [85]. Other co-operation of signaling pathways, blocking TGF-β and Wnt signaling and promoting FGF signaling, generates corneal epithelial cells from human iPSCs [61,76]. Supplementation of SB505124, IWP2, and bFGF in the induction medium produces high differentiation efficiency of hiPSCs into corneal epithelial cells that were positively stained with both specific corneal markers (CK12 and CK3) [61]. Treatment human ESCs with IWR1 (an inhibitor of Wnt signaling), A83-01, and bFGF can generate corneal epithelial cells expressed CK12 and CK3 [76] but the differentiated time is longer than Mikhailova et al. (2014) that may be caused by A83-01 slightly inhibited BMP signaling. Blocking Wnt signaling and activating BMP and FGF signaling by using RA, BMP4, and bFGF can generate corneal epithelial cells expressed CK3 [59]. Blocking Wnt signaling and activating BMP and EGF signaling via treatment human PSCs with RA, BMP4, and EGF can induce corneal epithelial-like cells [83]. A summary of signaling pathways involved in generation corneal epithelial cells is shown in Figure 1.

## 4. Corneal Epithelial Differentiation of Human MSCs

MSCs are widely used for cell therapies and regenerative medicine because of their great advantages of multipotent differentiation capacity, easy accessibility, and easy manipulation [88,89,90,91,92,93]. MSCs can be obtained from various adult tissues (e.g., bone marrow, adipose tissue, dental pulp, periodontal ligament, dermis, muscle) and fetal tissues (e.g., umbilical cord, placenta, amniotic fluid). According to the minimal criteria for defining MSCs by International Society for Cell & Gene Therapy (ISCT), MSCs are plastic-adherent cells with the capability of in vitro differentiation into adipocytes, chondrocytes, and osteoblasts. Moreover, MSCs must express CD73, CD90 and CD105 but not express CD11b, CD14, CD19, CD34, CD45, CD79a and HLA-DR [94]. Even though MSCs originate from mesoderm [95], MSCs are also able to differentiate into the cells of both endodermal and ectodermal lineages [96,97,98,99]. Likewise, MSCs can differentiate in vitro into corneal epithelial cells which are the cells in ectodermal linage.

To treat LSCD, transplantation of corneal or limbal epithelial cells are the common and effective procedures [100]. But the corneal tissues for isolating those cells are limited, thus there are attempts to use MSCs instead. Transplantations of MSCs for LSCD treatment in animal models have been being investigated and the results revealed satisfying outcomes. The investigated human MSCs were derived from various sources. Human bone marrow-derived MSCs (BM-MSCs) were grown on the amniotic membrane and transplanted in rats [7] or rabbits [101]. Human MSCs from immature dental pulps were prepared as cell sheets and transplanted in rabbits [102,103]. Human adipose tissue-derived MSCs (AT-MSCs) from orbital fat were directly transplanted by topical administration in mice [104]. Also, human AT-MSCs were directly transplanted by topical administration in rats [105] or grown on the amniotic membrane and transplanted in rabbits [106]. After transplantation, the epithelia were regenerated, the ocular surfaces were recovered, and corneal transparency was improved. The positive expression of CK3 or CK12 confirmed the terminal differentiation of the transplanted MSCs into corneal epithelial cells. Even though MSCs were transplanted without prior differentiation induction, the differentiation can be initiated because the cells at the transplantation sites can secrete signaling molecules directing them to become corneal epithelial cells [107,108,109]. Unfortunately, in some experiments, terminal differentiation was very low [104] or cannot be obtained [7]. Probably, the degree of differentiation after transplantation depends on the biological microenvironment at the transplantation sites which are difficult to be controlled [110,111,112].

In vitro differentiation induction can be achieved by using undefined induction medium and defined induction medium [113,114]. The undefined induction medium can be obtained from signaling molecules secreted by some appropriate cell types. The cells from cornea, both corneal cells and limbal cells, can provide signaling molecules regulating corneal epithelial differentiation [115,116,117]. Therefore, they are used to drive MSCs to differentiate into corneal epithelial cells in laboratories. In order to use signaling molecules from those cells, two methods are applicable; conditioned medium (CM) and co-culture system. In the first way, the corneal epithelial cells or limbal cells grown in culture containers will release inducing substances into the culture medium which can be harvested and used as CM. Once MSCs are cultured in the CM, MSCs will be induced to differentiate into corneal epithelial cells [118]. Another way is the co-culture system in which MSCs and the signal providing cells are cultured together in cell culture insert system. In such a culture system, one cell type is grown on the bottom surface of the culture container whereas another cell type is grown on the membrane surface of an equipment inserted in the same culture container [24,119,120]. The signaling molecules from one cell type can diffuse through the membrane pore to another cell type. In both strategies, the components in the medium cannot be defined and the signaling molecules playing roles in the differentiation induction cannot be specified.

Using CM as undefined induction medium was investigated in human AT-MSCs [118]. In this experiment, they compared the effects of the CM from human corneal epithelial cells and CM from human limbal fibroblasts on the corneal epithelial differentiation of AT-MSCs. Unfortunately, they found that AT-MSCs initially expressed CK3 and CK12. After AT-MSCs were cultured in the CMs, no significant difference was found in the expression of CK3. However, the expression of CK12 significantly increased after AT-MSCs were cultured in the CM from human corneal epithelial cells.

The co-culture system was applied in the differentiation of AT-MSCs [119], conjunctiva-derived MSCs (CJ-MSCs) [24], and exfoliated deciduous teeth-derived MSCs (EDT-MSCs) [120]. The MSCs in the experiments can undergo terminal corneal epithelial differentiation as shown by the results of gene expression or immunocytochemistry (ICC) of CK3 and CK12 markers. The details of the co-culture experiments are concluded in Table 1.

Using undefined induction medium from the aforementioned strategies need to culture signal providing cells in which there are some disadvantages; complex manipulation, expensive equipment in the culture insert system, and uncontrollable medium compositions. By contrast, defined induction media consist of known ingredient proportion and their ingredients can be adjusted to be suitable for specific cells. Moreover, defined induction media also can be used to investigate the effects of individual inducing molecules on the corneal epithelial differentiation. Various growth factors and bioactive molecules were added as ingredients of defined induction media. The defined induction media, which were used in the differentiation of human MSCs into corneal epithelial cells, and their results are summarized in Table 2.

In the differentiation of human CJ-MSCs by Soleimanifar et al. [24], they compared the differentiation outcome obtained from the co-culture as shown in Table 1 with the outcome obtained from the induction medium as shown in Table 2. According to their induction conditions, the co-culture offered much better differentiation outcome than the induction medium. However, the better outcome from co-culture possibly came from the effect of some bioactive molecules in the culture medium of the co-culture experiment. Because, when CnT-Prime medium was used in co-culture, the differentiation outcome from co-culture was poorer than the outcome from the induction medium. In the differentiation of human bone marrow-derived MSCs (BM-MSCs) by Katikireddy et al. [20], they determined the differentiation capacity between sorted SSEA4+ BM-MSCs and unsorted BM-MSCs in two-step induction in which different induction medium was used in each step. They found that sorted SSEA4+ BM-MSCs underwent corneal epithelial differentiation more than unsorted BM-MSCs. Nieto-Nicolau et al. [21] induced corneal epithelial differentiation of BM-MSCs by two types of defined induction media. The outcomes from the two media were different but, according to the results of CK3 and CK12 expressions, both media can induce the corneal epithelial differentiation.

In some experiments, combinations of differentiation methods were used and some stimulating conditions were applied in order to improve the results. In the differentiation of human adipose tissue-derived MSCs (AT-MSCs) by Bandeira et al. [8], induction media were used to initially induce AT-MSCs to become unspecific epithelial cells. Then, the initial induced cells were cultured as a cell sheet on fibrin gel before transplantation in rat LSCD model. The results revealed that initial induced AT-MSCs can undergo terminal differentiation in vivo into corneal epithelial cells (as shown by the expression of CK3 and CK12) and restored the corneal epithelium better than transplantation of undifferentiated AT-MSCs. In the differentiation of human BM-MSCs by Rohaina et al. [22], BM-MSCs were seeded on an amniotic membrane and co-cultured with mitomycin C-treated mouse fibroblast in an induction medium. Ten days after induction, the induced BM-MSCs formed cell sheet on the amniotic membrane and had gene expression of the determined markers (CK3, p63, C/EBPδ, β1-integrin) significantly higher than uninduced BM-MSCs. Moreover, Garzón et al. [23] can induce corneal epithelial differentiation of human Wharton’s jelly-derived MSCs (WJ-MSCs) to become corneal epithelium on their artificial cornea. Their artificial cornea was composed of two layers; stroma and epithelium. The stroma layer was fibrin–agarose gel seeded with human corneal keratocytes. The WJ-MSCs were seeded on the stroma layer. The constructs were submerged in an induction medium for 28 days. In such a condition, the differentiation of WJ-MSCs was induced by cytokines provided by the keratinocytes in the stroma layer and bioactive molecules in the induction medium. After that, the constructs were lifted up until the upper surface touched the air in order to induce stratification and terminal differentiation. By using this strategy, WJ-MSCs can differentiate into corneal epithelium and the obtained artificial cornea expressed marker profile similar to human cornea.

## 5. Corneal Epithelial Differentiation of Human PSCs

Both ESCs and iPSCs have pluripotent capacity to differentiate into many cell types. ESCs are isolated from inner cell mass of a blastocyst [17,122] while iPSCs are generated from embryonic or adult fibroblast cells [16,123]. These PSCs can be good candidates for treating LSCD. Transplantation of ESCs from animal and human successfully reconstructed ocular surface of host species [11,69,124,125,126]. Mouse ESCs could differentiate to be corneal epithelial-like cells (expressed CK12 and p63α), develop to form multilayer, heal porcine wounded cornea after transplantation, and maintain corneal marker expression [126]. Pax6 cDNA-transfected mouse ESCs showed expression of CK2, E-cadherin, and CD44 [69]. Moreover, they could expand to form three layers of epithelial cells on mouse damaged cornea [69]. Induced epithelial progenitor cells from mouse ESCs were successful generated and complete reconstructed mouse corneal surface after 24 h of transplantation [124]. Especially, cynomolgus monkey ESCs could be differentiated to be corneal epithelial-like cells which could form multiple cell layers on injured mouse cornea after transplantation [125]. Recently, transplantation of human ESCs has few publications, however, good results can be obtained. After six days of human ESCs transplantation onto human wounded cornea in vitro, these cells could expand on human Bowman’s membrane to produce one to four cell layers that expressed corneal markers (PAX6 and CK3) [127]. Noticeably, transplantation of epithelial cell sheet derived from human ESCs successfully recovered ocular surface of a LSCD rabbit and showed phenotype of limbal and corneal epithelial cells after eight weeks [11].

Although there is no publication about transplantation of iPSCs to treat LSCD eyes, these cells provide a valuable opportunity for vision therapy. Because iPSCs have similar differentiation capacity to ESCs but iPSCs can be generated from patients’ somatic cells so transplantation of autologous iPSCs can avoid immune rejection. Human iPSCs could be generate cornea organoids which expressed markers of adult corneal tissue [128] or displayed similar features of the developing cornea [129]. After explant cultures of minicorneas on denuded human amniotic membrane substrates for 10 days, the generated cell sheets expressed PAX6 and CK12 [128]. Human iPSC-organoid comprised of three cell types that express markers of corneal epithelium (CK3, p63), stroma (collagen type I and V), and endothelium (Collagen type VIII) [129].

To differentiate PSCs into corneal epithelial cells can use transgenic or non-transgenic methods. However, the transgenic method was only performed in mouse ESCs [69] while human PSCs only used non-transgenic method.

Non-transgenic method consists of the defined induction medium and CM. Several reports used CM from human limbal fibroblasts [25,130] or human limbal stromal cells [131] for induction of ESC/iPSC-derived corneal epithelial cells. Human limbal fibroblasts or stromal cells were isolated and expanded by subculture up to 10–15 passages [25,131]. These cells were treated with mitomycin C (MMC) for mitotic inactivation, then cultured in the corneal epithelial medium. The medium was changed daily and CM was collected every day until day even [25,130] or day 10 [131]. The detail component of corneal epithelial medium and the results are shown in Table 3. To collect a high population of derived corneal epithelial cells, the optimal time is ranged from one to two weeks depended on the cell types.

Differentiation of corneal epithelial cells from PSCs using the defined induction medium contains two or three steps (Table 3). Firstly, the ESCs/iPSCs are treated with induction media containing small molecules and/or growth factors involved in main signaling pathways that are necessary for corneal differentiation. The induction medium comprises defined and feeder-free medium (E6, RegES, mTeSR1, XF-Ko-SR) supplemented with small molecules (SB505124, IWP2 or IWR1, A83-01), bFGF with/without BMP4. Pluripotent cells will form embryonic bodies during suspension culture for about 4 days in the first step. After induction, pluripotent stem cells markers (Oct4, c-Myc, Nanog, Sox2) are significantly decreased while several transcription factors (PAX6, PITX2, BMP4, FOX1) regulated corneal development are increased [61]. Secondly, these cells are cultured in corneal epithelial medium (Cnt-30, CnT prime) or define-keratinocyte serum free medium (DKSFM) for further differentiation to corneal epithelial progenitor cells and maturation to corneal epithelial cells. Corneal epithelial progenitor cells show the markers of limbal stem cells such as p63, CK15, ABCG2, CK15, CK14 and the marker of proliferating cells (Ki67). Corneal epithelial cells express specific corneal markers (CK3, CK12) and other markers (CK15, CK14, and E-cadherin).

The efficiency of pluripotent stem cell-derived corneal epithelial cells is variable (Table 3). The highest efficiency is shown with 99% of cells positively expressed CK3 (H9 cell line) and 94% of cells expressed CK12 (RCM1 cell line) when the CM from limbal fibroblasts was used [130]. However, CK3 and CK12 protein expression results are very different from their mRNA expression results (CK3, CK12 mRNA expressed very low). Another report that also showed high efficiency of differentiation (70% CK12 positive, 30% CK3 positive) used RegES medium supplemented with two small molecules (IWP2, SB505124) and bFGF [61]. Moreover, they used a defined, serum-free, feeder free culture system that overcomes the disadvantage of using CM and co-culture system which had risks of pathogen transmission, immune response [132,133].

During the generation of pluripotent stem cell-derived corneal epithelial cells, collagen type IV is the most popular candidate for coating dish [25,61,83]. Collagen type IV, the main component of the basement membrane, is a form of extracellular matrix [134,135], supporting attachment and proliferation of limbal stem cells and corneal epithelial cells [25,136]. Another component of the basement membrane, laminin, is also used in combination with collagen type IV during differentiation into corneal epithelial cells [85,87].

Responsiveness of pluripotent stem cell lines are very different between cell lines [25,83,137]. Although the system for corneal epithelial cell generation is the same, the differentiated results are very variable. Some cell lines showed that they are not responsive for the induction of corneal epithelial cells (253G1, 201B7 [137], SB-Ad3 [83]). That maybe cause by the low activity of BMP signaling which could be improved by treatment with SB431542 [83].

## 6. Future Perspective

Even though the mechanism involving in the differentiation of stem cells into corneal epithelial cells has not yet been clearly clarified, many differentiation induction protocols have been proposed. Many publications reported the differentiation of stem cells into corneal epithelial cells but most of them are animal stem cell works. Only a limited number of publications studied the differentiation of human stem cells into corneal epithelial cells. There are differences in many aspects between the stem cells from animals and humans. Hence, the protocols developed from the studies on animal stem cells are probably not suitable to apply on human stem cells. Among the published reports of the corneal epithelial differentiation of human stem cells, various methods were used. Lack of comparison between the results obtained from each protocol causes unavailable of standard differentiation protocol. Therefore, the research to find good protocols suitable for each stem cell type is still required. This review concludes the published reports of the corneal epithelial differentiation of human MSCs and PSCs. According to the available reports, only small number of protocols have been investigated and the yields of successful differentiation are still not high. Therefore, protocols for inducing corneal epithelial differentiation have to be improved for better outcomes. Besides MSCs and PSCs, other kinds of human stem cells have also been investigated, especially the cells from ectoderm origin. Skin-derived precursor cells were induced corneal epithelial differentiation by a defined induction medium [138]. After the differentiation induction for 21 days, the result revealed incomplete differentiation because they expressed CK3 but did not express CK12. Oral mucosa epithelium is the source of adult cells which are widely used for corneal epithelium reconstruction [6,139,140,141,142]. The oral mucosa epithelial cells cultured on amniotic membranes offered satisfied outcomes after transplantation in patients in clinical trials. Umbilical cords from newborn babies are also the sources of cells for corneal epithelial regeneration. Umbilical cord lining epithelial cells cultured on human amniotic membrane expressed CK3 and CK12 [143] and can be used to treat patients with persistent epithelial defects [144].

Even though many kinds of human cells can be used for corneal epithelium regeneration, studies of corneal epithelial differentiation are still necessary to clarify the mechanism of differentiation. The obtained information from the investigations will provide useful knowledge in stem cell biology which is beneficial for medical applications in the future.

## Figures and Tables

**Table 1:** Corneal epithelial differentiation of human MSCs in the co-culture system.

Human MSCs	Signal Providing Cells	Culture Medium	Duration (Days)	Marker Expression at the Final Differentiation Stage	Reference
AT-MSCs	porcine limbal epithelial stem cells	DMEM FBS 10%	14	gene expression increase: CK3, CK12, p63 decrease: CD73, CD90, CD105, CK15, CK19, Cx43, ABCG2	[119]
CJ-MSCs	human corneal epithelial cells	SHEM medium FBS 5% DMSO 0.5% EGF 5 ng/mL Insulin 5 μg/mL Transferrin 5 μg/mL Sodium selenite 5 ng/mL Hydrocortisone 0.5 μg/mL Cholera toxin A 30 ng/mL	24	gene expression increase: CK3, CK8, CK12, CK14, CK15, ABCG2, DSC1, DSG1, NP63-α, Nestin, Involucrin ICC positive: CK3	[24]
EDT-MSCs	human corneal epithelial cells	DMEM/Ham F12 (1:1) FBS 5% DMSO 0.5% EGF 5 ng/mL	21	gene expression increase: CK3, CK19 ICC positive: CK3, CK19	[120]

Legend: DMEM, Dulbecco’s modified Eagle’s Medium; SHEM, supplemental hormonal epithelial medium; FBS, fetal bovine serum; DMSO, dimethyl sulfoxide.

**Table 2:** Corneal epithelial differentiation of human MSCs by defined induction media.

Human MSCs	Induction Medium	Duration (Days)	Marker Expression at the Final Differentiation Stage	Reference
CJ-MSCs	CnT-Prime 3D medium	24	gene expression increase: CK3, CK8, CK12, CK14, CK15, ABCG2, DSC1, DSG1, NP63-α, Nestin, Involucrin ICC positive: CK3	[24]
CJ-MSCs	DMEM: Ham’s F-12 (3:1) FBS 5% EGF 10 ng/mL Insulin 5 μg/mL Hydrocortisone 0.5 μg/mL Triiodothyronine 2 nM Adenine 2 nM	21	gene expression increase: CK3, CK8, CK12, DSC1, DSG1	[121]
BM-MSCs	Step 1 DMEM EGF 10 ng/mL BMP4 25 ng/mL All-trans retinoic acid 1 μM Step 2 DMEM: Ham’s F-12 (3:1) FBS 5% EGF 10 ng/mL Insulin 5 μg/mL Hydrocortisone 0.5 μg/mL Triiodothyronine 2 nM Adenine 2 nM	4 (step 1) and 12 (step 2)	gene expression increase: CK3, CK8, CK12, DSC1, DSG1 decrease: Oct4, Sox2, Nanog, Rex1, p63, ABCG2 ICC positive: CK3, CK8, CK12, CK14, CK15, β-integrin, E-cadherin negative: α-SMA	[20]
BM-MSCs	DMEM FBS 2% L-glutamine 2 mM All-trans retinoic acid 1 μM	7	gene expression increase: CK3, CK12, CK19, E-cad, ITGB1, Wnt-2, Snail decrease: Vimentin Protein expression increase: CK12, CK19, ITGB1, N-cad decrease: CK3, Vimentin, Snail, α-sma	[21]
BM-MSCs	SHEM/Ham’s F12 (2:1) FBS 2% DMSO 0.5% L-Glutamine 2 mM EGF 10 ng/mL Insulin 5 μg/mL Hydrocortisone 0.4 μg/mL Triiodothyronine 2 nM Adenine 0.18 mM	7	gene expression increase: CK3, CK12, ITGB1 decrease: CK19, E-cad, Vimentin, Wnt-2, Snail Protein expression increase: CK3, CK12, ITGB1 decrease: CK19, Vimentin, Snail, α-sma, N-cad	[21]

**Table 3:** Corneal epithelial differentiation of human PSCs.

Human Cell Line	Method	Step 1	Step 2	Step 3	Duration (Days)	ICC Result	Flow Cytometry/Cytospin Result	Gene Expression Result	Reference
ESCs (H1, hES-NCL1)	CM of MMC-treated limbal fibroblasts	3LG-DMEM/1F12 +hydrocortisone, insulin, adenine, EGF, tri-iodothyronine, cholera toxin	-		21	hES-NCL1: high positive with CK12, CK3/12, p63 on D7	Flow cytometry p63 highest on D6 hES-NCL1: CK3/12~55% highest on D6 H1: CK3/12~55% highest on D9	CK3 highest on D15 hES-NCL1: CK3+, CK12+ highest on D6 H1: CK3+++, CK12+++ highest on D9	[25]
iPSCs (L1B41, L1C51, 253G1, 201B7, L1B34)	Co-culture with MMC-treated PA6	GMEM +KSR, 2-mercaptoethanol			12–16 weeks	L1B41: CK12, CK3, PAX6, CK14		L1B41 high responsive: CK3, CK12: high express from week 8–12 Other cell lines low or non-responsive	[137]
ESCs (H9, H3, H14) iPSCs (6-9-9, 19-9-11)	Defined medium	DMEM/F12 +β-mercaptoethanol, DMSO, SU6656	DMEM/F12 +RA, BMP4, bFGF, β-mercaptoethanol	DKSFM	19	CK14, CK3, p63	Flow cytometry CK3 5%, CK14 90.7%		[59]
iPSCs	Defined medium	RegES medium +SB505124, IWP-2, bFGF	Cnt-30		44	D20 CK12, CK3, CK15, Ki67, Pax6	Cytospin on D44 p63 70%, CK3 30%, CK15 55%, CK12 70%	P63+++, CK15++, CK3+, and CK12+	[61]
ESCs (H9, RCM1)	CM from limbal fibroblasts	3LG-DMEM/1F12 +hydrocortisone, insulin, adenine, EGF, triiodothyronine, cholera toxin			21	-	Flow cytometry H9: CK3 99% on D21, high rate from D4–21 RCM1: CK12 94% on D21, high rate from D16–21	CK3, CK12: very low express, p63, CK19, ABCG2: high express on D16 and D21	[130]
iPSCs	CM from limbal stromal cells	EpiLife medium +human corneal growth supplement			21	CK3, CK12, p63 express from D7–21		CK3, CK12 high express on D14–21	[131]
iPSCs (UTA.045111.WT)	Defined medium	XF-Ko-SR D1: blebbistatin D2: SB505124, bFGF D3–4: BMP4	Cnt-30		21	CK14, CK15, p63, PAX6	Flow cytometry p63 71%		[85]
ESCs (H9) iPSCs (SBAd2, SBAd3)	Defined medium	mTeSR1 D1:Y27632	DMEM/F12 +BMP4/RA/EGF/LDN193189 or BMP4+RA+EGF or SB505124+IWP2 w/wo BMP4	CnT prime +10%FBS	20	CK12: not different on D20, CK3: lower express on D20 in SBAd3 group		BMP4+RA+EGF is the best SBAd2 &H9 responsive: CK3, CK12: high express SBAd3 non-responsive	[83]
ESCs (H1, H9, CT3, Envy (GFP^+^))	Defined medium	E6 +IWR1, A83-01, bFGF w/wo BMP4	E6		75	CK12, CK3	Flow cytometry CK3 55.6%, CK12 28.2%		[76]
ESCs (Regea 08/017, Regea11/013) iPSCs (UTA.04607.WT)	Defined medium	XF-Ko-SR D1: blebbistatin D2: SB505124, bFGF D3–4: BMP4	Cnt-30		24	D11: ABCG2+++, ∆Np63++, LGR5+++ D24: ABCG2+, ∆Np63+++, LGR5+, CK14+++, CK15++	Cytopsin: D11: ABCG2 62.4%, ∆Np63 23.2%, CD200 42.6% D24: ABCG2 1.8%, ∆Np63 54.3%, CK15 37%, CK14 56.2%		[87]
